# Hospital doctors’ and general practitioners’ perspectives of outpatient discharge processes in Australia: an interpretive approach

**DOI:** 10.1186/s12913-023-10221-3

**Published:** 2023-11-08

**Authors:** Edwin Kruys, Chiung-Jung (Jo) WU

**Affiliations:** 1 General Practice Liaison Unit, Sunshine Coast Hospital and Health Service, Birtinya, QLD4575 Australia; 2 School of Health, University of the Sunshine Coast, 1 Moreton Parade, Petrie, QLD 4502 Australia; 3 Royal Brisbane and Women’s Hospital (RBWH), Herston, QLD 4006 Australia

**Keywords:** Hospital doctors and general practitioners, Discharge planning, Barriers, Facilitators, Perspectives, Interviews

## Abstract

**Background:**

Unnecessary delays in patient discharge from hospital outpatient clinics have direct consequences for timely access of new patients and the length of outpatient waiting times. The aim of this study was to gain better understanding of hospital doctors’ and general practitioners’ perspectives of the barriers and facilitators when discharging from hospital outpatients to general practice.

**Methods:**

An interpretative approach incorporating semi-structured interviews with 15 participants enabled both hospital doctors and general practitioners to give their perspectives on hospital outpatient discharge processes.

**Results:**

Participants mentioned various system problems hampering discharge from hospital outpatient clinics to general practice, such as limitations of electronic communication tools, workforce and workload challenges, the absence of agreed discharge principles, and lack of benchmark data. Hospital clinicians may keep patients under their care out of a concern about lack of follow-up and an inability to escalate timely hospital care following discharge. Some hospital clinicians may have a personal preference to provide ongoing care in the outpatient setting. Other factors mentioned were insufficient supervision of junior doctors, a patient preference to remain under hospital care, and the ease of scheduling follow-up appointments.

An effective handover process requires protected time, a systematic approach, and a supportive clinical environment including user-friendly electronic communication and clinical handover tools. Several system improvements and models of care were suggested, such as agreed discharge processes, co-designed between hospitals and general practice. Recording and sharing outpatient discharge data may assist to inform and motivate hospital clinicians and support the training of junior doctors.

General practitioners participating in the study were prepared to provide continuation of care but require timely clinical management plans that can be applied in the community setting. A hospital re-entry pathway providing rapid access to outpatient hospital resources after discharge could act as a safety net and may be an alternative to the standard 12-month review in hospital outpatient clinics.

**Conclusion:**

Our study supports the barriers to discharge as mentioned in the literature and adds the perspectives of both hospital clinicians and general practitioners. Potential solutions were suggested including co-designed discharge policies, improved electronic communication tools and a rapid hospital review pathway following discharge.

## Background

One of the main pillars of the 2020 Council of Australian Governments (COAG) Health Agreement is to ensure timely access to health care for the community [1]. When hospital outpatient clinics retain patients unnecessarily this has direct consequences for timely access of new patients and hospital outpatient waiting times, which is a form of low-value care [2]. It has been argued that the traditional model of hospital specialist outpatient care may no longer be fit-for-purpose and places unnecessary financial and time costs on patients, clinicians, and the health system [2, 3]. Discharging patients from hospital outpatient clinics to general practice when they can be safely managed in primary care improves access to specialist outpatient clinics [4, 5]. Timely discharge is critical to ensure appropriate use of specialist services, streamline patient flow, and facilitate continuation of care in the community [6]. Continuity of care in general practice has a range of benefits, including greater patient satisfaction, increased medication adherence, reduced hospital usage and lower mortality rates [7].

The outpatient clinic discharge process is complex and dependent on many variables, including disease-based influences, clinicians’ attributes, patient factors, clinic organisation, presence of a discharge policy and availability of and trust in primary care [8, 9]. Scheduled hospital specialist follow-up appointments are not always necessary and often no interventions or treatment changes are initiated during these clinic appointments [10, 11]. A proportion of hospital outpatient visits are for routine and preventive care which could be performed in primary care to enable hospitals to adapt and respond to more complex cases [12, 13]. Implementing patient-focused discharge criteria reduces the number of unnecessary outpatient follow-up appointments, improves wait times and allows hospital specialists to use their expertise more efficiently [4, 14]. Often patients are seen by their general practitioner several times in between hospital outpatient visits, and it has been argued that there is a significant amount of duplication of care occurring. Discharging these patients when clinically appropriate may therefore not necessarily increase the general practice workload as it will improve access to patients referred by general practitioners for hospital specialist input in their care [15, 16].

Primary care doctors were satisfied with increased discharge from hospital specialist care, provided there was appropriate clinical handover [4]. Structured clinical handover reduces communication errors between health service organisations, and improves patient safety and care [17]. A review of hospital outpatient correspondence revealed that few outpatient letters documented clear management recommendations for the general practitioner to safely continue the care in the community, including advice regarding anticipated changes in the patient’s condition [15, 16]. Some argue there is room for improvement of discharge systems, so patients and their information can travel seamlessly across the interface between primary and secondary care [10, 12]. There is however limited evidence in the literature about the effective components of the discharge process from outpatient clinics to primary care.

### Aims

The aim of this study was to gain a better understanding of hospital doctors’ and general practitioners’ perspectives of the discharge processes from hospital outpatient clinics to general practice, especially the barriers and facilitators for discharge.

## Methods

### Research design

An interpretative approach was based on Heidegger's philosophy of “being in the world” [18], focused on understanding hospital doctors’ and general practitioners’ (GPs) perspectives of outpatient discharge process. This approach, incorporating semi-structured interviews with participants, enabled both hospital doctors and GPs to share their experiences with the outpatient discharge process [19]. 

### Settings and sample

This study was conducted in a 738-bed regional public hospital located in Queensland, Australia. Doctors across a diversity of specialties who met the inclusion criteria of hospital doctors regularly working in the participating hospital outpatients departments, general practice (GPs with Special Interest (GPSIs), aged over 18 years, can read and converse English, were invited to participated to the study.

### Data collection

After obtaining ethics approval, purposive sampling strategy was used to recruit potential participants. They were identified by contacting doctors with experience in the hospital outpatient discharge process as well as active community general practice, approached via internal emails. Data collection was carried out between September and November 2021. Data were collected through semi-structured interviews with each participant. The open-ended interviews were conducted by a trained research assistant who has experience in undertaking interviews in qualitative research under supervisors of the researchers. The interviewer was not a doctor, had no relationships with the hospital and the participants. To maintain consistency, all interviews were conducted by the same person. The questions were about their experience related to the discharge and clinical handover of patients from hospital outpatient clinics to general practice. The duration of each interview was 30–60 min, and the interview data were digitally recorded with consent of the participants. Interviews were conducted until saturation of data was reached when no new themes or essences emerged from the repeated data collection and analysis [20].

### Data analysis

On completion of the interview, data were transcribed into text files, followed by reading and re-reading the verbatim transcriptions [21] by the researchers (EK, C-JW). To maintain confidentiality, participant details were de-identified in the transcription process. Using content analysis, themes and categories were identified and modified as new information emerged [21]. The transcripts were each coded by two researchers. A list of codes was generated based on the emerging data and inter-coder reliability was confirmed between the two researchers and variations resolved by consensus.

### Ethics considerations

Full ethics approval has been obtained from Royal Brisbane and Women’s Hospital Human Research Ethics Committee (reference number: 65674). All ethical principles involving human research have been strictly adhered to, including obtaining individual doctor’s informed consent, ensuring voluntary participation without affecting their performance evaluation, privacy and confidentiality, data storage, and strict data accessibility.

## Results

A total 15 doctors were recruited of which 9 were hospital specialists representing 7 specialties, and 6 GPs. Hospital specialists and GPs believe not all follow-up appointments that take place in hospital outpatient clinics are necessary. Hospital specialists estimate that between 20–60% of patients seen in their outpatient clinics can be managed in primary care. GPs indicated that with appropriate clinical handover patients could often be followed up in general practice. Hospital specialists and GPs noted that patients are often not discharged back to GP-care when their condition has stabilised, and hospital specialist input is no longer required.

GP*5: "I see a lot of outpatient appointments that seem unnecessary, because they're monitoring things or they're doing things that we could do as GP if we just received better communication from the hospital."

HC**8: "Clinicians hold on to patients for too long and don't do the share care with GPs as we should be doing*."*

*GP: General Practitioner

**HC: Hospital Clinician

Multiple reasons were identified for not discharging patients, or delaying discharge, from outpatient clinics and these reasons were often interrelated (see table). Hospital specialists and GPs were well aware of the barriers and had potential solutions. Table 1 and Table 2 summarise the barriers and facilitators to effective discharge from hospital outpatient clinics to general practice.

**Table 1:** Barriers to effective discharge from hospital outpatient clinics to general practice

1. Clinical handover and communication to GP	• No/late clinical handover correspondence • Delays in clinical handover correspondence due to fluctuations in capacity/workload transcription services • Clinician delay in checking/signing off transcribed draft letters • Lack of time to write clinical handover correspondence • Communication by phone challenging due to clinical duties • Electronic hospital medical record unable to send messages to GPs • Lack of efficient interoperable secure electronic communication systems • Limited GP access to electronic hospital medical record • No hospital clinician access to GP medical records
2. Patient safety	• Concerns about lack of follow-up • Concerns about timely access to hospital care after discharge • Unsure if GPs are prepared to continue the care • Lack of trust in capacity/skills of other clinicians
3. Comprehensive care provision	• Preference to provide ongoing chronic disease management in hospital • The view that patients require ongoing hospital specialist care • Hospital clinician’s preference to dominate provision of care
4. Feasibility factor	• Discharge procedure is time-consuming (incl. patient reassurance, clinical handover documentation/ correspondence) • Scheduling follow-up appointments is easier than the more comprehensive discharge process • Follow-up appointments are often relatively straightforward and lighten the workload
5. Junior doctors	• Junior doctors are not always authorised or lack confidence to discharge • Insufficient capacity to supervise and guide junior doctors to support discharge • Junior doctors are copying follow-up practice of senior doctors
6. Patient factors	• Patients may be reluctant to be discharged • Some patients do not have a GP or are not engaged with their GP • Patients may prefer longer appointments in hospital
7. Workforce and workload	• Hospital workload does not facilitate discharge process • Concerns about increasing GP workload • GP shortage • General practice bulkbilling model
8. Discharge policies	• Discharge decisions are dependent on individual preferences of hospital clinicians • No consensus about discharge criteria or shared care processes • No consensus about required hospital outpatient follow-up care • Absence of discharge policies/procedures
9. Benchmark data	• No benchmark data available to compare • No patient/GP satisfaction data available • Audits not meaningful due to lack standards

**Table 2:** Facilitators to effective discharge from hospital outpatient clinics to general practice

1. Self-type/voice recognition software for clinical handover correspondence
2. (Co-designed) templated clinical handover correspondence for certain conditions/patient categories
3. Secure electronic two-way communication between hospital clinicians and GPs
4. Secure electronic GP advice models
5. Hospital consultant model of care instead of ongoing chronic care provision in hospital
6. Improving supervision and guidance of junior doctors to support discharge
7. Support staff alerting hospital clinicians about patients ready for discharge
8. Patient education about role of specialist vs GP and discharge process
9. Scheduling follow-up phone consultation prior to discharge
10. Providing GPs with clear, concise, and timely clinical handover management plans to facilitate care in the community
11. Introducing re-entry pathway as a safety net to escalate care rapidly following discharge if needed
12. Develop discharge principles
13. Collect and share benchmark discharge data

### Clinical handover and communication

Hospital specialists and GPs indicated that clinical handover and clinician’s access to key clinical information can be improved. GPs commented that they don’t always receive clinical handover correspondence and test results, or that it takes a long time before they receive communication from the hospital.

GP4: "If you don't get timely communication and the patient comes to see you, it's a very frustrating consultation because you don't know what's going on and you spend your time just trying to find out what's happened."

In the absence of clinical handover GPs sometimes have to make do with verbal information provided by patients which may be coloured by their interpretation and understanding of the consultation in the outpatient clinic.

GP1: "Patients will tell you what they've heard, but that's not always what the doctor's instructions are."

Hospital specialists acknowledged the variation in the time it takes send clinical handover correspondence to the GP.

An identified reason for communication delays was the fluctuating capacity and workload of transcription services responsible for typing dictated letters. Another reason mentioned was that it can take time before typed correspondence is checked and authorised by hospital clinicians, which is required before sending correspondence to GPs.

Hospital specialists frequently mentioned that a common barrier to providing clinical handover correspondence is time pressure. The workload in hospitals outpatient clinics often prevents them from writing clinical handover correspondence to the GP.

GP1: "If you want good correspondence from hospital clinicians, they actually need time to write the letters."

Some hospital specialists are not just using dictation/transcription services but are typing some or all the clinical handover correspondence at their own initiative or with a newly introduced letter typing and voice recognition software product, provided by the hospital. Some hospital clinicians have noticed this saves time while others found self-typing more time-consuming, although this was not always a reason to avoid it.

HC4: "It takes me longer to do it when I'm typing it myself, but I don't tend to write long letters, so I just get on with it and send it to the GPs, and that means they get the letter the same day that I saw the patient."

Templated clinical handover documents may assist hospital clinicians to provide clinical handover quicker, especially for routine procedures or conditions where the discharge advice is always more or less the same or requires only minor editing by clinicians. It was mentioned that providing written information to GPs according to a standard template also makes it easier for GPs to find the key information in the clinical handover document.

HC2: "I think templates are very helpful. You could have a drop-down list of things that you can add on and then you can potentially free text some of the more individualised options for that patient.”

HC9: "There should be a better template of what information needs to go to the GP. What is the reason you're communicating with them? And what do you want them to do with that information? It needs to be very short and sweet because GPs won't have time to read it."

It was mentioned that templates are not applicable for all patient cohorts. For example, in medical as opposed to surgical specialities the clinical handover information may be specific to a particular patient and needs to be individualised to a larger extent. GPs suggested that templates could be co-designed by hospital specialists and general practitioners to ensure it captures the information deemed important by all parties.

GPs and hospital specialists often need information from each other during or after a hospital care episode. GPs indicated they would appreciate the option to request and receive electronic written advice from hospital specialists either to avoid referrals and facilitate care in the community, or after discharge back to primary care, to support follow-up care.

GP4: “…if you have a simple question that they could clarify quite easily, then it would be nice to be able to just message a hospital specialist."

A frequently mentioned problem is the inability of hospital clinicians and GPs to efficiently communicate with each other via secure, electronic means. Lack of interoperable communication systems between hospitals and general practice was identified as one of the main *reasons.*

HC7: "Basically we have complete failure of real-time, asynchronous digital communication with primary care.”

HC9: "I guess, writing a letter to the GP, with the correct amount of data and monitor plan is probably the perfect way. The GP can go back and refer to that at any stage. The problem is that if the GP has questions, how do they ask the question to the hospital clinician?"

Part of the problem is that the Queensland public hospital electronic record does not have an inbuilt ability to communicate with other health providers including GPs, and third-party software is required to provide clinical handover. This is regarded by hospital clinicians as clunky as it often requires copying and pasting text between software programs or typing and dictating messages in other software programs.

HC5: "The hospital record is an electronic system. Information is typed in there, and it could be sent to the GP directly electronically, rather than going through the stepped process that involves some work from a clinician, some work from the typist, also some work from others, and then from the GP, and we are just unsure about how they received it. If there's an electronic system that we can just type in or transfer it, that will be ideal."

Hospital specialists frequently require information from GPs, ranging from clinical information to follow-up information after a hospital visit.

HC8: "Each time I send a patient to a GP, I would appreciate intermittent correspondence back, for example about a complex patient that I have seen in the clinic. I want to know that the patient is going to see their GP. Does the GP have comprehension of what we're trying to achieve, and are they able to follow through?"

Communication via the phone can be challenging for clinicians as both hospital specialists and GPs are often unable to take calls when they are providing patient care. However, in cases where they can talk to each other via the phone, the experiences are often positive.

Another barrier to sharing care is the inability to access electronic medical records across primary and secondary care. The National My Health Record, which can contain various clinical summary documents, is not widely used. The Queensland public hospital service uses an alternative database called the Health Provider Portal, which contains hospital pathology and imaging reports as well as hospital outpatient correspondence and inpatient discharge summaries (provided they have been created). This database does not have a notification system to inform GPs that information has been uploaded and not all GPs have requested access.

### Patient safety

Patient safety concerns are often mentioned as a reason not to discharge. Some hospital clinicians were concerned that the process of handing over care could lead to clinical omissions including lack of follow-up.

HC8: "I'm concerned that something will get missed. Therefore, I book the patient every year to come back because I am not trusting anyone else to follow up on that one thing. And it may mean that I need to do an [investigation] on a patient every two or three years, but I'm concerned that if I don't see them every year then someone else might miss that."

Some hospital specialists have experienced that a GP did not act on their recommendations in the discharge plan, which strengthened their belief that patients are better off remaining under the care of the hospital.

Long waiting lists can be a disincentive for discharge as clinicians and patients are concerned that patients may not get back into the clinic in a timely manner if their condition worsens following discharge.

HC9: "It's really hard to get the patient back into our clinic so if we discharge them, and then the GP wants to send them back to us because they've got a new problem, or they've got a flare up of their problem, then they become a new referral and might be on the waitlist, and that could be anywhere from 3 to 12 months."

Hospital specialists with a long waitlist therefore may find it more difficult to discharge patients compared to those with short wait lists, which was labelled a self-perpetuating problem as not discharging adds to the waitlist problem.

Furthermore, hospital specialists stated they are sometimes unsure if GPs are prepared to accept and continue the care of patients following discharge. Lack of trust in the capacity or skills of other clinicians responsible for the follow-up also plays a role.

### Wanting to provide comprehensive long-term care

Some hospital clinicians may be reluctant to discharge patients because they are of the opinion that they are in the best position to provide the care their patients need or prefer to keep control over the care.

HC8: "There is always that element of, no one does it better than yourself - type thing, which is a bit of a weird thing but I know what I'm looking for so I should probably then follow it up."

HC9: "We have the assumption, rightly or wrongly, that we're the only ones who are capable of looking after the patients. We tend to hold on to them, rather than send them back to the GP."

At the same time hospital clinicians appear very aware of the challenges this creates with regards to the capacity to see new patients. It was mentioned that a hospital specialist ‘consultant model of care’ also has benefits.

HC2 “[Our specialty] takes a very holistic approach, which in the good old days was a great thing to be able to do but I think more problematic now we are in this fiscal environment with the demand being so high and knowing that there's all these patients out there on the waitlist who need to be seen.”

HC4: "It comes down to your personality, and actually wanting to solve all their problems instead of, for example, an NHS philosophy of saying, ‘we just need specialist care for this problem, the GP is very capable, they can manage the rest.’”

Because clinicians are seeing many patients with chronic conditions there may be a tendency to keep them in the hospital system.

HC4: "A lot of the conditions we deal with are chronic. So, where do you draw the line? It's a bit vague sometimes. However, we always have a front of mind about how we discharge. Some of my colleagues are better than others about doing it."

HC8: "There is an old school thought that if you've got an [organ-specific] problem, you need to be seen forever. And so, a lot of my colleagues keep these patients in their clinic.”

GPs commented that long-term hospital specialist care is not always necessary.

GP4: "A lot of the time the specialist takes ownership, where they probably don't need to. They could probably pass it on to the GP.”

### Convenience factor

The discharge process entails several steps requiring time and effort, such as explaining to the patient that their care will be safely handed over to the GP and writing clinical handover correspondence, including a management plan for the GP and the patient. Scheduling a follow-up appointment is less time-consuming than organising discharge and a handover to the GP. Relatively straightforward follow-up appointments also appear to lighten the workload in the usually hectic and often overbooked outpatient clinics.

HC9: "Some clinicians are just keeping the patients that are stable because those patients are easy.”

GP4: “Sometimes it's just easier for the specialists to organise investigations and then arrange for a follow up, as opposed to communicating that with the GP, making sure they understand that that needs to be done and why."

### Junior doctors not discharging

It was mentioned that junior doctors may not have permission to or are not confident to discharge patients. Insufficient capacity to supervise and guide junior doctors in the discharge process appears to play a role.

HC7: "The system is so overstretched and overloaded, and our ability is limited to supervise the junior staff to ensure that they follow best practice to discharge people, according to the principles where it's safe. We just can't. And suddenly the whole system just churns on further and further, it is so broken that it becomes stagnant."

Some junior doctors may copy the habits from their seniors in regularly reviewing patients with stable conditions in the outpatient clinic.

HC8: "If they've seen the consultant bringing the patient back every year for the last three years, they are not all of a sudden going do anything different. And then some patients get into a registrar clinic, and they'll be stuck there for another two or three years. I think sometimes if the staff identify the patients being seen by the registrar every year for two or three years, they try to put the appointment back into the consultant clinic."

In general, improving the supervision of junior doctors and registrars was seen as a way to improve clinical handover and increase the discharge rate. It was also mentioned that supporting clinicians with flagging patients who may be ready for discharge would be helpful.

### Patient factors

Hospital specialists indicated that patients can be reluctant to be discharged and often need to be reassured that discharge is appropriate and safe, especially when patients have been waiting a long time to get a hospital appointment. Patients sometimes advise hospital clinicians that they feel safer remaining under the care of a hospital specialist. Occasionally GPs also need to be reassured that discharge is safe.

HC8: "That process is a real challenge in terms of how to make it so that the patients are comfortable to be discharged, and that the GP is understanding that they have to be discharged, and that people don't think that you're just being reckless.”

Sometimes patients remain worried about their health condition.

HC6: "Often we're treating people's anxiety that there might be something wrong, as opposed to actually being something wrong."

Some hospital clinicians overcome patient anxiety about discharge by scheduling a follow-up phone appointment and if there are no concerns at that point in time, they discharge the patient.

Occasionally patients believe that their GP may not have the skills or knowledge to continue to look after their condition. Sometimes patients advise that they do not have a regular GP or are not engaged with their GP. Hospital clinicians also have the impression that some patients prefer the longer consults offered in hospital.

Although patients are not routinely informed about the discharge process and clinical handover to the GP, some hospital specialists make an effort to explain the reason for discharge, and their role versus the role of the GP, to reassure patients. They often mention explicitly that their GP can always re-refer in case of any problems following discharge. Hospital clinicians and GPs agree about the need for a pathway to escalate care back to the hospital following outpatient discharge, when needed. This pathway could act as a ‘safety net’.

GP1: "A re-entry process is a blessing for the patient and makes it easier for the GP to get a patient back in."

HC3: "I would support a rapid re-entry for people who are well known to a specific department to avoid them having to be re- referred and go through that whole process again.”

### Workforce and workload

Both primary care and hospitals are facing cyclical workforce challenges. Hospital clinicians identified the shortage of GPs and lack of bulkbilling opportunities as a barrier for patients to follow-up care in the community. At the same time, hospital clinicians acknowledged that the bulkbilling model does not always facilitate comprehensive care in the community.

HC8: "If it's a predominantly bulkbilling practice, then the GP may not have as much time for the clinic appointment in which case those patients will often say, ‘I don't get any time, I can't do this’. That becomes somewhat awkward because I don't want to become their GP."

Some hospital clinicians were concerned about increasing the GP workload when discharging more patients, and therefore keep patients under their care.

HC3: "The problem is that regular reviews then need to be done by the GP, which might increase their workload. There needs to be a balance between the specialist department following up, versus overwhelming the GPs.

HC5: "It has to be good for the workforce both ways, because we have to distribute the workload appropriately, we don't want to be lopsided.”

GPs appeared to be prepared to take over the care provided there is a clear management plan handed over to them from hospital clinicians.

GP1: "We can do anything they want with clear, concise, timely instructions on what they want us to do. What the next step or two is, you know, try something for a month, and if no better add this, or change to that etc."

Hospital outpatient clinics are often being overwhelmed and find it challenging to keep up with the demand on outpatient services.

HC2: "I think more and more, we're under a lot of pressure with inadequate resources to see the patient in a timely fashion. There's also the issue of appointment slots being overbooked. We have less time to spend on an adequate assessment and doing an adequate letter to the GP."

Some hospital clinicians believed that despite the challenges there is a need to better facilitate discharge when clinically appropriate.

HC9: "I don't necessarily think more doctors is the answer. I think we just need to get better at how we're doing things. And I need to be able to empower my colleagues to feel safe, that we can discharge patients and there is a re-entry pathway. It's changing people's behaviour to make that happen, which is really difficult."

### Absence of policies

Hospital clinicians mentioned that there is no consensus about when to discharge patients, when to share care with GPs or which patient groups should continue to be booked for outpatient follow-up appointments. This decision-making process appears dependent on individual assessments of the treating hospital clinicians.

HC2: "There's no formal set of criteria as to when someone gets discharged, it's just sort of, you know, there's some personal preference behind it."

There are at present no uniform local, statewide or national hospital discharge principles or discipline-specific guidelines to support the outpatient discharge or continuation of care process, and some clinicians noted that there is a need for more consistency.

HC6: " If we had a pathway that would be really good. So, we could say, it is hospital policy, you haven't had [specific signs or symptoms] in six months so we're discharging you. If you develop more problems the GP can refer you back.”

HC8: "So, when you want things to run efficiently you need to have a very consistent approach. If a junior medical officer or a locum or I see someone else's patient, you have a consistent approach, and it doesn't make any difference because we're always doing the same thing."

HC9: "And if it was almost like a KPI that would put a lot more pressure on my colleagues to adopt that. But at the moment they don't feel obliged."

### Absence of benchmarking

Hospital specialists indicated there are no benchmark data about discharge rates, and no information about patient or GP satisfaction after discharge. Because of the lack of uniform or agreed processes there is no standard to compare against and audits are complicated and often not meaningful.

HC1: "For our specialty there is no national benchmark on what the average discharge rates should be for clinics. We have no data to help drive us, so we can only do what we can."

HC1: "How do other hospitals run their clinics? How do they do their discharge processes? What are their outpatient discharge rates? And how do they collaborate with the GPs? How efficient am I as a clinician, you know? I've been doing this clinic for so long and generally I feel most patients are happy, but once they go back to the GPs, what do they actually say?"

Implementing discharge principles was seen as a valuable tool to assist timely discharge, to set a standard and to assist with data collection, benchmarking, and quality improvement.

HC8: "I don't disagree that people can have their own approaches, but it should be within a couple of standard deviations of the normal. At the moment we have no way of auditing, because there is no consistency, so we don't know who's doing what and how they're doing it. How do you build a process around something that you don't know?

HC9: "I'd like to be able to get onto a dashboard really easily and have that data. I do think it needs to be part of a KPI where we actually are constantly being reminded."

## Discussion

Our findings are consistent with the barriers to discharge stated in the literature. The additional perspectives of both hospital clinicians and general practitioners provide further information such as the importance of clinical handover and communication tools for hospital clinicians, alternatives to face-to-face hospital appointments, a rapid hospital review pathway following discharge, and the potential benefits of codesigning elements of the discharge process.

An effective handover process requires protected time, a systematic approach, and a supportive clinical environment. Both hospital clinicians and general practitioners indicated there is room to improve clinical handover between primary care and secondary care. Clinical handover is an important requirement to facilitate safe discharge from hospital outpatient services [16]. It is more than the transfer of information; it is about maintaining continuity of care [22]. Giving clinicians user-friendly, interoperable electronic tools to improve bi-directional communication appears a system enabler for effective discharge. These approaches should include hospital clinicians’ preference for dictation via transcription services, voice-recognition dictation, self-typing, and customisable templated letters [23].

Our findings are consistent with literature reporting on the importance of improving two-way communication which may alleviate the patient safety concerns and trust issues raised around clinical handover to primary care and follow up in the community setting [24]. GP-advice models and joint (phone or telehealth) appointments with GPs were seen by some as solutions to improve communication and reduce unnecessary face-to-face appointment with patients in hospitals. GPs appear prepared to take over the care but require timely clinical management plans that can be applied in the community setting.

Given the large number of people involved in the discharge process, it appears that improving the discharge process, including implementing discharge principles, requires involvement and education of all stakeholders, including hospital specialists, junior doctors, nurses, administrative staff, GPs and patients, to ensure all parties are aware of and comfortable with the process. Some participants argued that discharge principles and policies should be co-designed between hospitals and general practice to improve usability, uptake, and adherence by all stakeholders [6, 25].

Our finding of the absence of benchmarking data in the participating hospital suggested that recording and sharing benchmarked outpatient clinic discharge data may assist to inform and motivate hospital clinicians and support the training of junior doctors. Current statewide metrics required to be reported and monitored by Queensland public Hospital and Health Services include: “*Number of long waits at census by category and by specialty; Number of booked and un-booked at-risk patients who are due for treatment over the following 30, 90 and 365 days to ensure there is sufficient capacity to manage existing waiting lists as well as additional referral trends and patient review lists within services* “ (p.47) [26].

It was mentioned that a rapid re-entry pathway after discharge would service the category of patients with health conditions that are not an emergency but also cannot wait for more than several weeks to be seen. It would act as a safety net and could also be an alternative to the standard 12-month review in outpatient clinics; instead, the patient could be discharged and be re-referred in case of clinical deterioration related to the same clinical problem. This would especially benefit specialties with long wait lists. The re-referral could result in a face-to-face appointment or management advice to the GP and patient for example via phone or secure electronic communication [27]. Participants mentioned that facilitating discharge from outpatient clinics could increase the burden on GPs; this may however be offset by an increased capacity for outpatient clinics to see patients who are on a waitlist and managed in primary care. Some outpatient clinics appear to already have similar systems in place.

There were different views on the extent of responsibility hospital clinicians should assume when a patient is referred to them. The ‘consultant model of care’ appears to facilitate earlier discharge when clinically appropriate, as opposed to taking over chronic disease management from primary care. This may require a shift in thinking about the outpatient clinic model, away from long-term care and towards more episodic care provision, supported by effective clinical handover mechanisms and strong ties with primary care. Safe continuation of care following discharge requires an adequate workforce capacity of experienced medical professionals with an appropriate skill-mix in general practice [28].

### Limitations and strengths

We acknowledge our qualitative approach has a degree of subjectivity, and the interpretative approach is an inherently subjective process. Although care was taken to minimise this, for example through posing neutral, open-ended interview questions and the dual coding of the transcripts, it is likely that this has influenced our results to a degree.

The community GPs in our study were also working part-time as GPs with a Special Interest (GPSIs) in various hospital specialty outpatient clinics. This meant that they were knowledgeable about the hospital discharge and clinical handover processes and therefore provided valuable insights, but they may not necessarily represent the typical community GP. Also, as they have hands-on involvement with the hospital discharge process, their views may have been biased to an extent. Another limitation is acknowledged, as the interview was conducted in a regional hospital, it may not be appropriate to generalise findings to other states or territories. Although the study shared common limitations of a qualitative study, strengths of this study have contributed to an in-depth understanding of hospital doctors’ and GPs’ perspectives on the discharge process.

### Future research

Future research could consider barriers and facilitators from hospital doctors and GPs in other contexts, with additional key performance indicators to clarify and refine future quantitative research in the area.

## Conclusion

Our study confirms the barriers to discharge as mentioned in the literature, and ads the perspectives of both hospital clinicians and general practitioners. Potential solutions were suggested including co-designed discharge policies, improved communication tools and a rapid hospital review pathway following discharge.

## Data Availability

Data sharing is not applicable to this article as no datasets were generated or analysed during the current study.
